# An LED-Driven AuNPs-PDMS Microfluidic Chip and Integrated Device for the Detection of Digital Loop-Mediated Isothermal DNA Amplification

**DOI:** 10.3390/mi11020177

**Published:** 2020-02-08

**Authors:** Zengming Zhang, Shuhao Zhao, Fei Hu, Guangpu Yang, Juan Li, Hui Tian, Niancai Peng

**Affiliations:** State Key Laboratory for Manufacturing Systems Engineering, Xi’an Jiaotong University, Xi’an, 710054, Shaanxi, China; zhangzengming@stu.xjtu.edu.cn (Z.Z.); zsh.qq.com@stu.xjtu.edu.cn (S.Z.); hufei0701@xjtu.edu.cn (F.H.); st170558@stud.uni-stuttgart.de (G.Y.); lj2931012643@163.com (J.L.); tianhui2016@stu.xjtu.edu.cn (H.T.)

**Keywords:** digital LAMP, gold nanoparticles, integrated device, DNA quantification

## Abstract

The sensitive quantification of low-abundance nucleic acids holds importance for a range of clinical applications and biological studies. In this study, we describe a facile microfluidic chip for absolute DNA quantifications based on the digital loop-mediated isothermal amplification (digital LAMP) method. This microfluidic chip integrates a cross-flow channel for droplet generation with a micro-cavity for droplet tiling. DNA templates in the LAMP reagent were divided into ~20,000 water-in-oil droplets at the cross-flow channel. The droplets were then tiled in the micro-cavity for isothermal amplification and fluorescent detection. Different from the existing polydimethylsiloxane (PDMS) microfluidic chips, this study incorporates gold nanoparticles (AuNPs) into PDMS substrate through silica coating and dodecanol modification. The digital LAMP chip prepared by AuNPs-PDMS combines the benefits of the microstructure manufacturing performance of PDMS with the light-to-heat conversion advantages of AuNPs. Upon illumination with a near infrared (NIR) LED, the droplets were stably and efficiently heated by the AuNPs in PDMS. We further introduce an integrated device with a NIR heating unit and a fluorescent detection unit. The system could detect HBV (hepatitis B virus)-DNA at a concentration of 1 × 10^1^ to 1 × 10^4^ copies/μL. The LED-driven digital LAMP chip and the integrated device; therefore, demonstrate high accuracy and excellent performance for the absolute quantification of low-abundance nucleic acids, showing the advantages of integration, miniaturization, cost, and power consumption.

## 1. Introduction

Owing to its superior performance over real-time nucleic acid amplification technology (qNAAT) in terms of accuracy, specificity, and reproducibility, digital nucleic acid amplification technology (dNAAT) is widely used in low-abundance nucleic acid quantification for the diagnosis of cancer, viruses, and bacterial infections [1,2]. For dNAAT-based nucleic acid detection, DNA samples are divided into thousands of microdroplets or microchambers, which are subsequently amplified at specific temperatures. DNA concentrations can be accurately measured through the combination of endpoint fluorescence detection and Poisson probability models [3]. According to the temperatures required for nucleic acid amplification, dNAAT can be divided into digital polymerase chain reaction (dPCR) and digital isothermal amplification technology (dIAT) [4]. As a branch of dIAT, digital loop-mediated isothermal amplification (digital LAMP) permits nucleic acid amplification under isothermal conditions, thus eliminating the need for the complex thermocycling procedures used in dPCR [5,6].

Studies on dNAAT have focused on sample segmentation using innovative microfluidic chips [7,8,9]. Although the microfluidic chips for dNAAT have emerged endlessly, most dNAAT instrument systems still require more than three sets of auxiliary devices, including sample segmentation unit, heating unit and fluorescence detection unit. As an example, in commercial dNAAT systems based on microdroplets such as QX 200 (Bio-rad), DNA samples must be transferred between a microdroplet generator, a thermal cycler, a sealing instrument, and a flow fluorescence detection device [2,10]. In the QuantStudio 3D (Thermo Fisher Scientific) dNAAT system, DNA samples are dispersed into the microchambers of a silicon-based chip using a sample loading device, and incubated in a Peltier heater prior to fluorescence imaging [11]. These systems perform to a high level, but rely on multiple auxiliary devices leading to complex operating procedure and high-power usage. The future of dNAAT development; therefore, focuses on portability, integration, and miniaturization [12,13,14].

In the course of NAAT, a series of heating schemes based on Peltier [15], near infrared (NIR) laser [16], acoustic waves [17], and other mechanisms were proposed and combined with fluorescence detection technology, thereby promoting the emergence of integrated qNAAT instruments including qPCR devices. Among these heating schemes, the NIR heating demonstrates the advantages of large heating area, fast speed, and easy integration with fluorescence detection unit. Since 2015, gold nanostructures with strong surface plasmon resonance effect have been gradually applied in the field of NAAT research. Due to its excellent photothermal efficiency, the introduction of gold nanostructures allowed photothermal NAAT to get rid of the reliance on costly laser equipment, and even realized LED-driven NAAT [18]. Lee and colleagues employed a polymethylmethacrylate (PMMA) cavity covered with two thin gold nanofilms to evenly absorb light to heat the PCR mixture, and established a heating device based on a 3 W LED arrays [19,20]. Roche and coworkers introduced gold nanorods into the PCR reactions to achieve ultra-fast and real-time plasmonic qPCR under the illumination of a 2 W laser, which completed 30 thermal cycles in 54 s [21]. Weizmann’s group extended their photothermal system based on gold bipyramids to isothermal nucleic acid amplification and restriction enzyme digestion [22]. In the current development of qNAAT towards dNAAT, the introduction of LED-driven heating technology into dNAAT is of great value in promoting the integration and miniaturization of related instruments. However, these heating methods that performed well in qNAAT are not directly applicable to dNAAT, which is a complex technique that requires the integration of sample segmentation and heating. For example, the opaque gold films employed by Lee led to challenges for subsequent fluorescence detection, and the PMMA substrate is not the ideal material for dNAAT microfluidic chip due to its reliance on complex and expensive processing methods. If gold nanoparticles (AuNPs) are mixed into microchambers or microdroplets, it is difficult to ensure the uniform distribution of AuNPs. In addition, in Roche and Weizmann’s research, AuNPs have a certain inhibitory effect on DNA amplification. Although they have proposed some methods to reduce the inhibition effect of AuNPs for DNA amplification, it is not clear whether these methods can be applied to dNAAT reagent, which is a biochemical system with lower tolerance to external additives than qNAAT [23]. To employ gold nanostructures for dNAAT heating, not only the heating performance itself, but also sample segmentation, chip manufacturing and subsequent optical detection should be taken into consideration systematically.

In this study, to overcome the limitations of these methods, we proposed an AuNPs-PDMS microfluidic chip that realizes microdroplet-based sample segmentation and surface plasmon resonance heating-based nucleic acid amplification. Due to its high performance for microstructure manufacturing and thermostability, PDMS has been used in microfluidic chips for microdroplet dNAAT (ddNAAT) and microchamber dNAAT (cdNAAT). The introduction of gold nanoparticles into the PDMS substrate imparts the PDMS with a compact and integrated heating function under illumination. The AuNPs-PDMS described herein were obtained through the mixing of silica-coated and dodecanol-modified AuNPs-ethanol with PDMS prepolymers and then evaporating the ethanol. The advantage of this strategy is that the content and morphology of the AuNPs can be independently regulated with no loss of manufacturing capability and bonding performance of PDMS. In addition, the AuNPs dispersed in the PDMS substrate will not inhibit nucleic acid amplification.

We designed and prepared patterned AuNPs-PDMS films based on an SU-8 mold, and bonded the AuNPs-PDMS film between a bottom and a top glass substrate to fabricate a “glass–PDMS–glass” sandwich digital LAMP chip. The digital LAMP chip integrates a cross microchannel for droplet generation and a microcavity for LAMP amplification and fluorescence detection. Driven by two syringe pumps, the digital LAMP mixture was evenly divided into multiple microdroplets and tiled in the AuNPs-PDMS microcavity. Under the illumination of a near-infrared LED (808 nm), the AuNPs-doped PDMS achieved uniform and stable heating, leading to the simultaneous amplification of target DNA in the droplets. In addition, we established an integrated device that combined NIR-LED heating and fluorescent detection. As a proof of concept, we evaluated the performance of the chip and integrated device with serial dilutions of hepatitis B virus (HBV) DNA, which demonstrated an accurate detection of low-abundance nucleic acids. To the best of our knowledge, this is the first study to achieve an absolute quantification of nucleic acids based on the photothermal effects of gold nanoparticle. The integrated device for NIR LED heating and fluorescence detection has the advantage of low costs, a compact size, and low energy consumption. This highlights the potential of the system to promote the development of dNAAT towards portability, integration, and miniaturization.

## 2. Experimental Design

### 2.1. Materials and Reagents

Hepatitis B Virus (HBV) DNA templates were obtained from Nucleic Acid Quantitative Assay Kits (P101, Tianlong, China) and their initial concentrations were confirmed by qPCR instrument (Gentier 96E, Tianlong, China). DNA template was stored at -20 °C prior to use. Digital LAMP primers were purchased from Sangon Biolotech Co., Ltd (Shanghai, China) according to HBV sequence. The LAMP primers were as follows: 

Forward outer primer (F3): 5-TCCTCACAATACCGCAGAGT-3; backward outer primer (B3): 5-GCAGCAGGATGAAGAGGAAT-3; forward interior primer (FIP): 5-GTTGGGGACTGCGAATTTT-GGCTTTTTAGACTCGTGGTGGACTTCT3; reverse interior primer (BIP): 5-TCACTCACCAACC-TCCTGTCCTTTTTAAAACGCCGC-AGACACAT-3.

Oil phase reagent (HFE7500, 3M, containing 2% surfactant) were purchased from Bio-Rad (Hercules, USA). Fluorescent dye including calcein and manganese chloride were purchased from Sigma-Aldrich (St. Louis, MO, USA). Bst DNA polymerase, ThermoPol® buffer, betaine, and deoxyribonucleotide triphosphate (dNTP) were purchased from Sangon Biolotech (Shanghai, China). The components of the 20 μL digital LAMP reagent system used herein are shown in Table 1. It should be noted that before adding the DNA template to the LAMP reagent, the HBV-DNA template needs to be quantified by standard qPCR method and then serially ten-fold diluted, so that the initial template concentration range in the LAMP system is 1 × 10^1^ to 1 × 10^4^ copies/μL.

PDMS prepolymer (SYLGARD 184 A) and curing agent (SYLGARD 184 B) were purchased from Dow Corning Inc (Midland, USA). All other chemicals were obtained commercially and used without purification.

### 2.2. Preparation of AuNPs and AuNPs–PDMS

**Figure 1:**
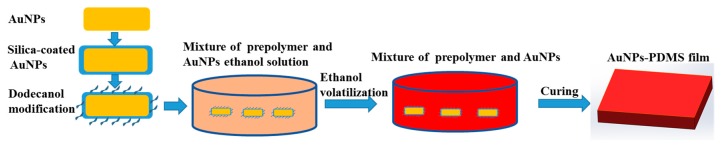
Schematic diagram of the AuNPs-PDMS preparation.

AuNPs prepared in aqueous reagent have a tendency to agglomerate in organic PDMS [24]. Herein, we adopted a method of silica coating and dodecanol modification to make AuNPs dispersed in ethanol that are miscible with PDMS prepolymer, as shown in Figure 1. As a class of AuNPs with tunable absorption peaks, gold nanorods were synthesized using the seed-mediated growth method [25]. Briefly, seed solutions were prepared through the mixing of hexadecyl trimethyl ammonium bromide (CTAB) solution (10 mL, 0.1 M) and HAuCl_4_ (0.085 mL, 0.028 M) with fresh NaBH_4_ (0.07 mL, 0.1 M). For the growth of the gold nanorods, 0.3 mL seed solution was added to CTAB (12 mL, 0.1 M), sodium oleate (18 mL, 0.013 M), HAuCl_4_ (0.5 mL, 0.028 M), HCl (1 mL, 0.1 M), AgNO_3_ (0.32~0.37 mL, 0.01 M), and ascorbic acid (0.05 mL, 0.1 M). Following incubation at 30 °C for 24 h, the newly-produced AuNPs colloids were centrifuged at 10,000 rpm for 30 min, decanted, and resuspended in 30 mL of 1 mM CTAB to decrease free CTAB and sodium oleate levels. To enhance the stability of the gold nanorods at high temperatures, silica-coated gold nanoparticles (AuNPs@SiO_2_) were synthesized using the Stober method. NaOH (0.1 M) was added dropwise to adjust the pH of AuNPs to 10.4~11.0. Next, 0.1 mL of tetraethyl orthosilicate (TEOS) was added for 1 h with shaking and left for 12 h for static growth. A layer of silica was successfully coated onto the surface of the nanorods which were then centrifuged at 10,000 rpm for 20 min and decanted. The AuNPs were mixed with dodecanol to a total volume of 30 mL. Next, 1 g of C_7_H_8_O_3_S was added and after ultrasonic dispersion for 10 min, the solution was transferred to a high-temperature reactor and incubated at 70 °C for 3 h. The solution was then centrifuged at 11,000 rpm for 30 min, decanted, and resuspended in 30 mL of ethanol. Following ethanol washing and centrifugation, aqueous AuNPs were concentrated in 3 mL of ethanol. Of note, dodecanol modifications could reduce the hydrophilic hydroxyl groups on the silica shell, enhancing the lipophilicity and dispersion of the AuNPs in PDMS.

AuNPs-PDMS was prepared as shown in Figure 1. The AuNPs-ethanol solution was added to PDMS prepolymers, stirred, and then heated in a 70 °C ventilated dryer for ≥ 2 h to fully evaporate the ethanol. AuNP-PDMS films were obtained after mixing the AuNPs-doped PDMS prepolymers with curing agent at a weight ratio of 10:1, and then curing at 90 °C for 1 h. To study the photothermal characteristics of AuNPs-PDMS film, we prepared 5 AuNPs-PDMS films at a range of AuNPs concentrations. For these 5 sample films, the volume of the concentrated AuNP solution incorporated into 3 g of PDMS before evaporating were 1, 2, 3, 4, and 5 mL, respectively. The relative mass of the AuNPs in the AuNPs-PDMS films were estimated as 0.031%, 0.062%, 0.093%, 0.124%, and 0.155%, respectively. It should be noted in advance that the AuNPs content for the digital LAMP chips was 0.093%.

### 2.3. Digital LAMP Chip Design and Fabrication 

**Figure 2:**
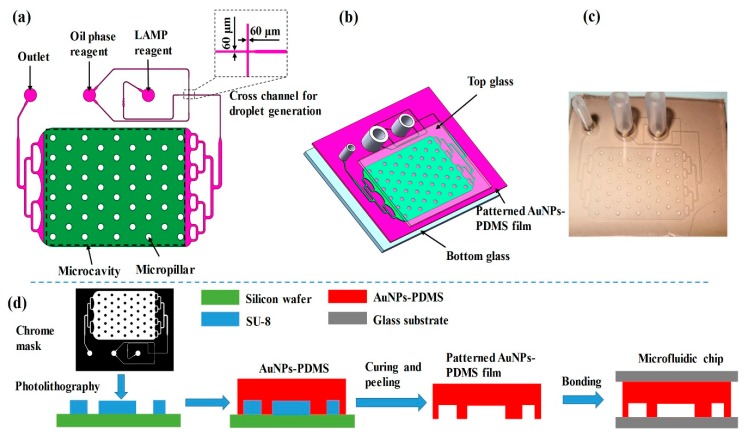
Schematic of the AuNPs-PDMS chip. (**a**) Top view. (**b**) Explosive view. (**c**) The appearance of digital LAMP chip. (**d**) Simplified fabrication process of the chip.

A schematic of the digital LAMP microfluidic chip is shown in Figure 2 and was designed using Auto CAD. The chip consists of a cross microchannel for droplet generation and a microcavity for droplet tiling. Based on the principle of flow focusing, nucleic acid samples and LAMP reagents were divided and wrapped into multiple water-in-oil droplets. These water-in-oil droplets were transferred and tiled in the microcavity through a three-stage branch flow channel. The integrated chip has a small footprint with a length of 43 mm, a width of 32 mm, and a height of 8 mm. The width of droplet-generating microchannel is 60 μm (Figure 2a), which is a dimension that can be easily prepared by PDMS. The microcavity has a length of 20 mm, a width of 15 mm, a height of 0.1 mm, and can collect and tile ~20,000 droplets with a diameter of ~100 μm. To avoid the collapse of the microcavity, 50 micro-pillars with diameters of 1 mm were designed.

The digital LAMP microfluidic chip was fabricated based on soft lithography processes and sandwich assembly. As shown in Figure 2b,d, a layer of SU-8 2050 negative thick photoresist (PR, MicroChem Corp., Newton, MA, USA) with a thickness of 100 μm was first spun onto a silicon substrate and followed by a soft bake process. A standard lithography process with an exposure dose equal to 230 mJ/cm^2^ was performed to copy the pattern of the chrome mask onto the SU-8 photoresist. The SU-8 development process was finished by immersing the exposed substrates into a developer solution (MicroChem Corp., Newton, MA, USA) and using ultrasonic agitation to obtain well-defined SU-8 structures. After treatment with octafluorocyclobutane (C_4_F_8_) for 3 min to facilitate demolding, the SU-8 mold was ready for the preparation of patterned PDMS film. Similar to the preparation of the AuNPs-PDMS film, the AuNPs-doped PDMS prepolymer (with 0.093% AuNPs) and curing reagent were mixed at a weight ratio of 10:1, respectively, and poured onto the SU-8 mold. Following degassing and heating in a vacuum desiccator at 90 °C for 1 h, the microchannel of the SU-8 mold was replicated on the AuNPs-PDMS film. The patterned AuNP-PDMS film had a thickness of 1.5 mm and was peeled off from the silicon wafer, installed with three joints, and bonded to a top and a bottom glass through oxygen plasma treatment. The sandwich assembly avoided the thermal evaporation of droplets. To ensure reliability, drops of pure PDMS were applied onto the edge of the microfluidic chip and heated for curing. After rinsing with fluorosilane and drying, the chips were ready for digital LAMP experiments (Figure 2c). 

### 2.4. Design and Establishment of Integrated Device for NIR Heating and Fluorescence Detection

**Figure 3:**
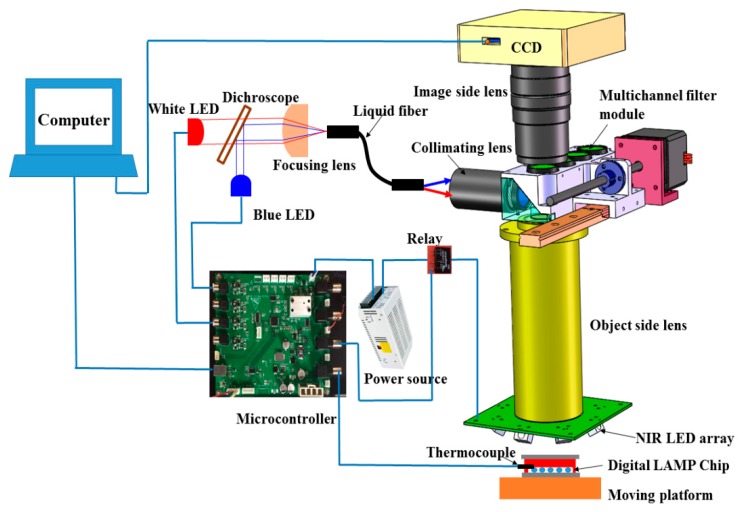
Overview of the integrated device for NIR heating and fluorescence detection.

To integrate the heating and fluorescence detection unit for digital LAMP into a single device, we designed and fabricated an integrated prototype as shown in Figure 3. The overall dimensions of the device were 210 mm in length, 150 mm in width, and 330 mm in height, which was more compact than the existing commercial instrument. 

The NIR heating unit consisted of a circularly arranged NIR LED array (24 V, 12 W, peak wavelength at 808 nm, Vanch Photoelectric, Inc., Shanghai, China) to provide the NIR radiation for heating. Type-K thermocoupling (5SC-TT-K-40-36, Omega Engineering) was used for temperature monitoring, and a switching mode power supply (24 V, Weihua Electronics, Inc., Xi’an, China). The NIR-LED array has a small footprint with a length of 58 mm, a width of 58 mm, and a height of 22 mm. The distance between the chip and the object side lens is 27 mm. The circularly-arranged LED array consisted of 8 LEDs in series illuminated on the microcavity zone of the chip at an angle of incidence of 45°. The circularly arranged LED array made the NIR irradiation more uniform, and multiple LEDs ensured sufficient NIR heating power and large heating area. The temperature controlled system was implemented using a microcontroller based on ARM (STM32F103RET6). The NIR LED array was powered through a 24 V power supply controlled by a TTL (transistor transistor logic)-controlled relay (CMX60D10, Crydom Co., San Diego, CA, USA). The TTL line was actuated at 1000 Hz with the duty cycle controlled by the program built into the microcontroller. During thermal incubation, the microcontroller showed an output of 3.3 V to close the TTL-controlled relay to illuminate the NIR LED. Once the temperature from the thermocoupler exceeded 62.5 °C, the microcontroller led to an output of 0 V TTL and disconnected the power supply of the NIR LED, prompting the temperature of the digital LAMP reagent to return to ~62 °C. When the temperature dropped below 61.5 °C, the reverse operation was performed.

The fluorescence detection unit consisted of a CCD camera (C11440-50U, 6.97 × 5.23 mm, Hamamatsu, Japan), a customized object side lens (149 mm focal length), an image side lens (MVL100M1, 100 mm focal length, Thorlabs), a multichannel filter module, a blue LED (M470L3, 470nm, 3.3 V, 0.76 W, Thorlabs), and a white LED (MCWHL5, 0.38 W, 3.3V, Thorlabs). Light from the blue LED or white LED passed through a long-pass dichroscope (DMLP470R, 25 × 36 mm, Thorlabs), a focusing lens (ACL2520U-B, Thorlabs), a liquid optical fiber (16 mm diameter, NA 0.5, Chunhui, Inc., Nanjing, China), a collimating lens (65-553, Edmund), a multichannel filter module, and a customized object side lens and illuminated the microcavity zone. The filters and dichroic mirror mounted in the multichannel filter module were: FF01-495/28-25 (Semrock) for excitation, FF01-525/39-25 (Semrock) for fluorescence detection, and FF497-Di01-25×37 (Semrock) to split the beams of excited and omitted light. Using the drive of the linear motor, the multichannel filter module could switch from fluorescent detection to brightfield imaging. According to the focal length of the object-side and image-lens, the magnification of the optical system was 0.67, and the size of a single imaging zone was 10.4 × 7.8 mm. Since the size of micro cavity was 20 × 15 mm, four shots were required to obtain the complete images of all the droplets. 

### 2.5. Image Analysis

Under white LED, the CCD camera obtained bright-field images, which were analyzed to count the total number of droplets. Fluorescence images of the droplets were acquired under the action of blue LED and filter components, and were then used to distinguish whether the droplet was positive or negative for the target DNA. Image processing was performed using Image J and MATLAB. Briefly, according to the algorithms such as image filtering, local threshold processing, watershed-based image segmentation, and particle statistics [26], both the number of total and positive droplets were individually counted. According to the Poisson probability model, the average DNA copy number were calculated using the following equation:

Concentration = −ln (1−*N*_p_/*N*_t_)/*V*_d_ (1)

Where *N***_t_** represents the total number of droplets in the digital LAMP chip, *N*_p_ represents the number of positive droplets, *V*_d_ represents the droplet volume [7].

## 3. Results and Discussion

### 3.1. Characterization of AuNPs and AuNPs-PDMS Films

**Figure 4:**
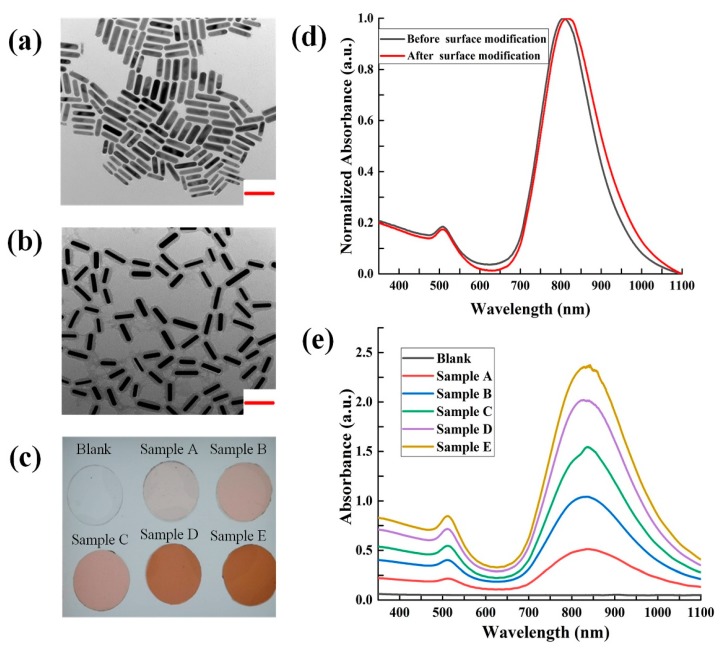
Characterization of the AuNPs and AuNPs-PDMS films. (**a**) TEM images of the AuNPs. (**b**) TEM images of the silica coated AuNPs. (**c**) Images of the AuNPs-doped and blank PDMS films. (**d**) UV-Visible absorption spectra of the AuNPs. (**e**) UV-Visible absorption spectra of the AuNPs-PDMS films. The scale bar is 100 nm. AuNPs concentrations in sample films A~E are 0.031%~0.155%.

To avoid photo-bleaching of the NAAT fluorescent dye and probe during NIR thermal amplification, the NIR LED was set to 808 nm [21]. The longitudinal absorption peak of gold nanorods were controlled near to 808 nm to achieve efficient photothermal conversion. Based on this condition, the gold nanorods with corresponding longitudinal absorption peaks were prepared. We used silica coating and surface modifications using dodecanol to improve the lipophilicity and dispersibility of the nanorods in organic solvents including ethanol and PDMS prepolymers. TEM (transmission electron microscope) images of the AuNPs are shown in Figure 4a. An average length of 80 nm and an average diameter of 20 nm were observed. TEM images of the silica coated AuNPs are shown in Figure 4b, and indicate that a 15 nm thick silica layer was successfully wrapped on the surface of the AuNPs. Upon comparison of the UV-Visible absorption spectra of unmodified AuNPs in water and modified AuNPs in ethanol (Figure 4d), the longitudinal plasmon resonance band redshifted from 810 nm to 816 nm. This reflected the increase in the refractive index of the medium around the AuNPs [27]. 

Five AuNPs-PDMS films, which were doped with different concentrations of AuNPs, and a blank PDMS film were sectioned into circular pieces with a diameter of 12 mm and a thickness of 1.5 mm (Figure 4c). To characterize the light absorption properties of the AuNPs-PDMS, UV-Vis absorption spectroscopy were obtained (Figure 4e). Due to the plasma effects of the gold nanoparticles, AuNPs-PDMS films showed distinct absorbance peaks at ~512 and ~828 nm, whilst the blank PDMS showed no absorbance peak in the UV and visible region. Compared to the UV-Vis absorption spectra and color of the sample films of A–E, as the content of the AuNPs increased, the corresponding absorption intensity increased. Since the refractive index of the PDMS (n = 1.42) exceeded that of water (n = 1.33) and ethanol (n = 1.36), the transverse and longitudinal absorption peaks of the AuNPs–PDMS appeared as a red-shift [27]. In addition, excluding the transverse and longitudinal absorption peaks, no other absorption peaks in the UV-Visible absorption spectra were observed. This suggested that the AuNPs did not agglomerate in PDMS and ethanol, further illustrating the effectiveness of the adapted preparation method for AuNPs-PDMS film. 

### 3.2. Photothermal Performance

**Figure 5:**
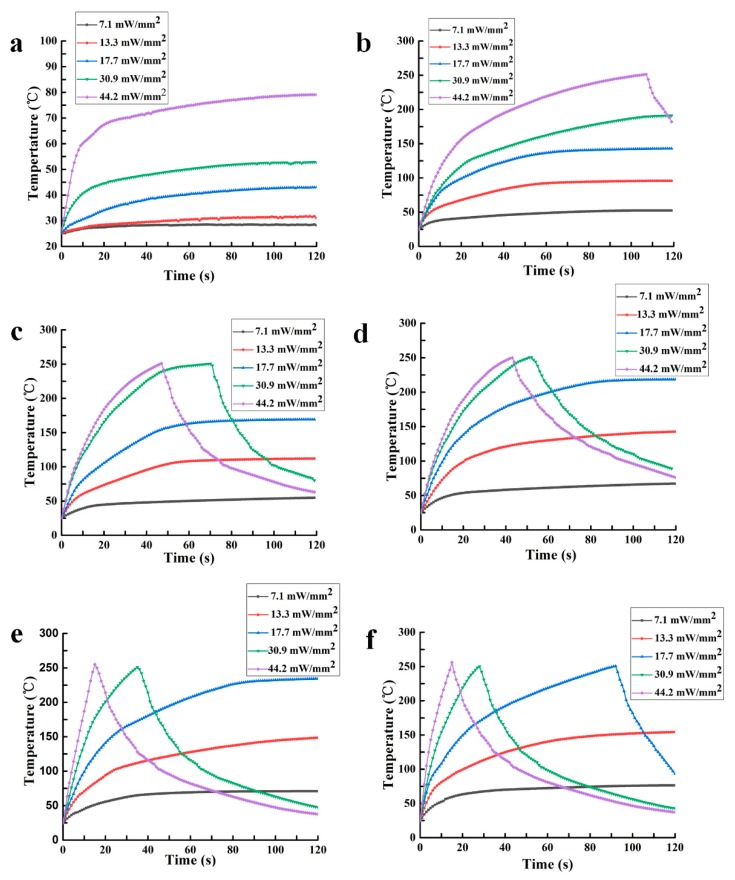
Temperature curves of the sample films under different radiation intensities. (**a**) Blank PDMS films without AuNPs. (**b**) Sample film A—0.031% AuNPs. (**c**) Sample film B—0.062% AuNPs. (**d**) Sample film C—0.093% AuNPs. (**e**) Sample film D—0.124% AuNPs. (**f**) Sample film E—0.155% AuNPs.

The aim of this study was to produce a digital LAMP chip based on the photothermal effect of the AuNPs-PDMS films. It was; therefore, necessary to study the photothermal performance of the AuNPs-PDMS films, particularly regarding the equilibrium temperature and heating rates. To simplify the protocols, the AuNPs-PDMS film was bonded between two glasses to simulate the increase in temperature of the LAMP chip. We used a thermocoupling instrument (5SC-TT-K-40-36, Omega Engineering) to record temperature changes within the PDMS films containing different levels of gold nanoparticles under the illumination of a multimode fiber semiconductor laser (808 nm, 0~5 W, Leirui Laser, Inc., Changchun, China). The spot diameter of the laser on the AuNPs-PDMS film was 12 mm, and the laser power was adjusted to 0.8, 1.5, 2, 3.5, and 5 W, in turn. Each film was irradiated under different NIR laser powers for ~120 s. Temperature measurement experiments were performed three times for each film. The representative temperature rise curves are shown in Figure 5. As the PDMS burns at 250 °C, the laser was switched off at this temperature to allow heat dissipation. For comparison, the temperature curve of blank PDMS films were also recorded (Figure 5a). At an irradiation of 7.1 mW/mm^2^, blank PDMS only warmed by 3.5 °C, and the temperature increased to 73 °C under 44.2 mW/mm^2^. Figure 5b–f demonstrates that once the AuNPs were doped into the PDMS film, the AuNPs-PDMS film could be heated above 54 °C at 7.1 mW/mm^2^. This is significantly different from blank PDMS, and demonstrates excellent photothermal effect of AuNPs-PDMS. When the relative content of the AuNPs exceeded 0.093% under an illumination intensity of 7.1 mW/mm^2^, the temperature could rise above 64 °C. However, under the illumination intensity of 30.9 mW/mm^2^, blank PDMS films barely reached 52 °C, indicating that the introduction of the AuNPs reduced the optical power of the photothermal LAMP by ≥ 77%. In addition, when the radiation intensity exceeded 13.3 mW/mm^2^, PDMS resulted with an AuNPs content ≥ 0.062% that could be heated from 25 to 94 °C within 40 s. These data suggest that the AuNPs-PDMS film meets the heating requirements of LAMP, and can be used for PCR that requires fast temperature cycling between 60 and 94 °C. 

**Figure 6:**
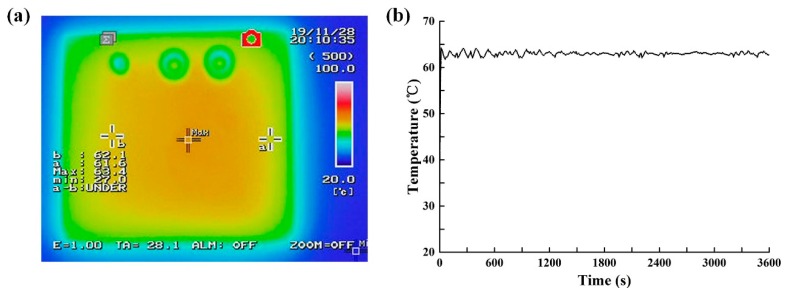
Chip heating performance. (**a**) Temperature field distribution. (**b**) Temperature stability.

Since the entire area of the heating zone is 20 × 15 mm, the temperature uniformity should be considered. Prior to the assembly of the integrated device, the experimental system was built based on the NIR LED heating components described in Figure 3, and the temperature field of LAMP chip was then recorded using an infrared thermal camera. As shown in Figure 6a, the temperature of the chip ranged from 61.6 to 63.4 °C. The temperature at the center of chip exceeded that of the edge. This occurred due to the larger temperature difference compared to the external environment, leading to rapid heat dissipation at the edge. In addition, the radiation of the NIR LED array was not completely uniform, which also caused a non-uniform distribution of the temperature field. Future studies should focus on improving the uniformity of the temperature distribution by improving the NIR source and optimizing the heat dissipation of the chip. The LAMP experiments performed well between 60 to 64 °C, meaning that all droplets in the chip can be heated to the temperature required for LAMP amplification [28].

In addition, the entire process of LAMP amplification is as long as 45 min, so the temperature stability during this process needs to be evaluated. We utilized a thermocouple attached to the central surface of the digital LAMP chip to record the temperature profile within 60 min under the control system and the NIR-LED irradiation, as shown in Figure 6b. The temperature profile indicated that the chip temperature can reach ~62 °C within 17 s and then fluctuated slightly between 61.8 and 63.6 °C, and this is an acceptable fluctuation range for LAMP amplification.

Compared with the contact-type hot-plate heating method widely used in commercial instruments, this non-contact LED-driven heating solution no longer requires the hot cover for reducing the temperature difference between the hot plate and the chip [29]. This is because AuNPs can efficiently and directly convert the energy from the NIR-LED source into heat of the PDMS film. At the same time, thanks to the uniform illumination distribution from multiple LEDs, this LED-driven heating solution also does not require a thermally-conductive aluminum block used in the hot-plate solution for evenly distributing the temperature field. In addition, those complicated and bulky aluminum blocks and hot cover can cause difficulties in automatic chip switching during fluorescence detection, while the non-contact heating method can make the mobile platforms easier to implement. Therefore, the LED-driven heating scheme proposed in this study is an effective solution to realize the miniaturization and integration of digital LAMP instruments.

### 3.3. Performance Characterization of Digital LAMP Chip

**Figure 7:**
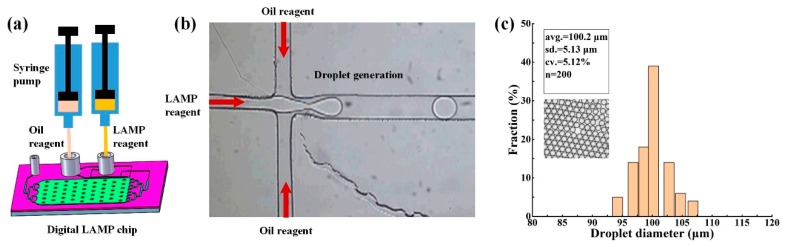
Schematic illustration and characterization of droplet generation. (**a**) Device schematic. (**b**) Instant image during droplet generation. (**c**) The droplet size distribution.

The core functions of the integrated digital LAMP chip should include sample segmentation in addition to isothermal heating. In this study, DNA sample segmentation was achieved by generating water-in-oil droplets based on the principle of flow focusing [5,30]. Driven by two syringe pumps, the water-phase reagent (LAMP reaction solution) and the oil-phase reagent (HFE7500) meet at a cross microchannel of the digital LAMP chip, as shown in Figure 7a,b. The LAMP reagent was then sheared and pinched off by the continuous oil-phase to form dispersed microdroplets, into which the DNA templates were divided. During droplet generation, the flow rate of the oil phase and the LAMP reagent was 12 and 6 μL/min, respectively, so it takes ~4 min to complete sample segmentation of 20 μL of LAMP reagent. The droplet size profile is of great value for characterizing the sample segmentation performance of the microfluidic chip, because the variation in size of the droplets can bias the Poisson-based calculations of template quantification. In order to characterize the size profile of the microdroplets generated by the digital LAMP chip, we measured the diameter of 200 droplets using a microscope and the Image J software. The average diameter was measured to be 100.2 μm with a coefficient of variation of 5.12% (Figure 7c), such uniformity in droplet size is similar to that of a previous study [5,31].

### 3.4. Digital LAMP Operation and Verification

**Figure 8:**
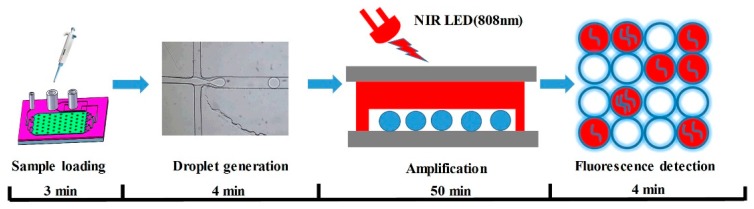
The operation process of digital LAMP assay.

DNA quantification based on the digital LAMP method in this study requires the steps shown in Figure 8, including sample loading, droplet generation, isothermal amplification, and fluorescence detection. Prior to digital LAMP assays, DNA template, LAMP reagents, and the oil phase reagents were freshly prepared. The volume of the LAMP mixture was 20 μL, as outlined in Table 1. As described in Section 3.3, the DNA template was divided into a large number of droplets with average diameter of 100.2 μm, and then the droplets were tiled in the microcavity. Following droplet generation, the inlets and outlets of the chips were sealed with plastic caps, and the chips were then loaded on the moving platform of the integrated device (Figure 3). After attaching a thermocouple to the chip and turning on the power and control system, the DNA amplification driven by LED can be started. After 45 min of amplification, the microcontrol system turned off the NIR-LED and turned on the blue LED for fluorescence detection, and then the CCD acquired the fluorescence images. Because the signal of the negative droplet in the fluorescence image is weak, in order to facilitate the statistics of the total number of droplets, the bright-field images of the chip were also obtained under the action of white LED and corresponding filter mode. It should be noted that the exposure times for fluorescent and bright-field images are 1 s and 40 μs, respectively. In addition, during optical detection, the chip needs to be moved four times so that the CCD acquired the fluorescence and bright-field images of all the droplets tiled in the chip.

In this study, the entire operation process took only 61 min (Figure 8), which saved nearly half of the time compared to traditional qPCR. Compared with commercial dNAAT instruments such as QX 200 (Bio-rad) and QuantStudio 3D (Thermo Fisher Scientific), the microfluidic chip reported herein integrates functions including sample segmentation, heating, and droplets tiling for detection, and avoids sample loss and contamination during multi-step sample transfer. Meanwhile, the AuNPs with high photothermal performance incorporated in the chip realizes stable heating under the irradiation of low-power and low-cost LEDs, thereby enabling facile integration of the heating unit and the fluorescence detection unit. Therefore, the chip and integrated device proposed in this paper can promote the development of the dNAAT system towards miniaturization, portability, and economy.

The sensitive quantification of low-abundance nucleic acids hold great value for clinical applications. To evaluate the accuracy of the digital LAMP chip and the integrated device, ten-fold serial dilutions of HBV DNA stocks were prepared at four orders of magnitude from 1 × 10^1^ to 1 × 10^4^ copies/μL, deemed a low nucleic acid concentration range. Using the digital LAMP chip and integrated device, we performed nucleic acid quantifications on the HBV samples according to the operation process mentioned above. Each experiment was performed in quadruplicate to ensure reproducibility. 

**Figure 9:**
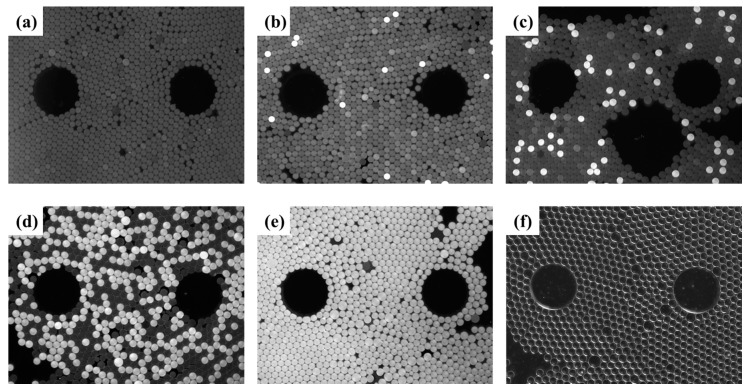
Digital LAMP assessments as varying concentrations of HBV DNA. (**a**) Negative control. (**b**–**e**) Digital LAMP fluorescent images (partially shown) for serially diluted samples of HBV DNA ranging from 1 × 10^1^~1 × 10^4^ copies/μL. (**f**) Representative bright-field image (partially shown).

After droplet generation and amplification based on our chip and device, we obtained fluorescence images and bright-field images, as shown in Figure 9. The gray value of the negative and positive droplets were compared and analyzed using Image J software. The results showed that the gray value of the positive droplets was about 2.6~3.5 times that of the negative droplets. This phenomenon indicates that DNA has indeed undergone amplification. Because the calcein used in this study is a fluorescent dye whose fluorescence can be quenched by Mn^2+^. The pyrophosphate ions caused by DNA amplification can react with Mn^2+^ to form intolerant salts, thereby reducing the concentration of free Mn^2+^ in reagent, leading to an increase in fluorescent signal. As seen in Figure 9, the droplet size was relatively uniform, indicating that most did not break or fuse during DNA amplification. Comparing and analyzing the a~e panels in Figure 9, we can find that as the concentration of template DNA increased, the number of bright positive droplets increased. 

**Figure 10:**
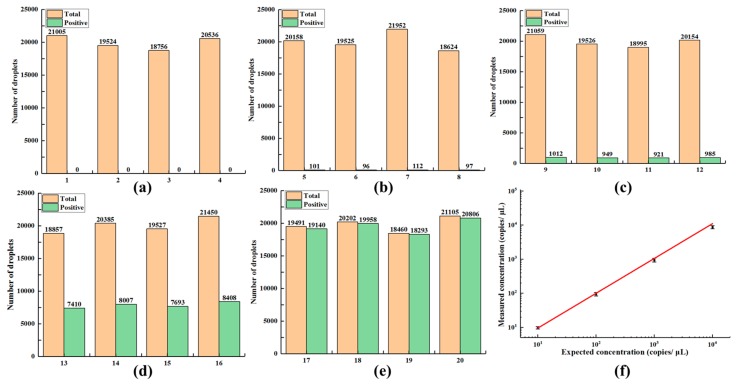
Verification results of digital LAMP. (**a**) Statistics of droplets for negative control. (**b**–**e**) Droplet statistics for serially-diluted DNA samples. (**f**) Linear relationship between measured values (copies/μL) by the digital LAMP chip and the expected copy number per reaction. Four tests were performed for each data point.

For digital LAMP assessments, the ideal conditions are that each droplet contains only one or zero template DNA molecules, and the number of positive droplets directly reflects the number of DNA molecules. However, for actual detections, the DNA is not evenly divided and the sample concentration are insufficiently diluted, with some droplets containing more than one DNA molecule. It is; therefore, necessary to combine the Poisson probability model to correct the DNA concentrations of the original samples, as described in Section 2.5. We used Image J and MATLAB to analyze fluorescence and bright-field images, and calculate the proportion of positive droplets by counting the number of positive droplets in fluorescence images and the number of total droplets in bright-field images, as shown in Figure 10. The average ratios of positive droplets in those serially-diluted DNA samples after amplification were 0.51%, 4.85%, 39.29%, and 98.93%, respectively, while there were no positive droplets in negative control. As shown in Figure 10f, the measured concentrations of serial-ten-fold diluted HBV-DNA samples were well correlated with the expected concentrations (*R*^2^ = 0.9985), which demonstrates the feasibility of the developed digital LAMP chip and device for quantification of low-abundance nucleic acids. At the same time, the batch-to-batch variation of the replicates of DNA samples with the same concentration was small, which reflected the good repeatability of digital LAMP-based DNA detection.

## 4. Conclusions

In summary, we developed a digital LAMP chip integrated with DNA amplification heating function. The chips consisted of AuNPs-doped PDMS which enabled the PDMS to have higher light-to-heat conversion efficiency that retained the original advantages of the microstructure manufacturing process. The AuNPs content was varied from 0.031% to 0.155% to study the UV-Vis absorption spectra and photothermal characteristics of the AuNPs-PDMS films. The results showed that AuNPs with a relative mass of 0.093% could heat the PDMS films to above 62 °C under an NIR radiation of 7.1 mW/mm^2^, and could reduce the power of the NIR light source by ≥ 77%. We designed and prepared a digital LAMP chip based on this AuNPs-PDMS composite for droplet generation and photothermal amplification. Using flow focusing, approximately 20,000 water-in-oil droplets were generated and tiled in the microfluidic chip that consisted of AuNPs-PDMS. Utilizing the photothermal performance of the digital LAMP chip, we further developed an integrated device with an NIR heating unit and fluorescence detection unit. The integrated device realized LED-driven microfluidic heating and imaging-based fluorescence detection, demonstrating low cost, low levels of power consumption, high integration, and a reduced requirement for auxiliary equipment for digital LAMP experiments.

## Figures and Tables

**Table 1:** The components of the digital LAMP reagent.

Components	Concentration	Volume	Remark
ddH_2_O	/	7 μL	/
Betaine	0.8 M	2.7 μL	/
ThermoPol® buffer	10×	2 μL	/
dNTP mix	10 mM	0.7 μL	2.5 mM of each of the four dNTPs
Forward outer primers	10 μM	0.7 μL	/
Backward outer primers	10 μM	0.7 μL	/
Forward interior primers	20 μM	2 μL	/
Reverse interior primers	20 μM	2 μL	/
Bst DNA polymerase	8000 U/mL	1 μL	/
DNA template	/	0.7 μL	/
Fluorescent dye	/	1 μL	0.5 μM calcein and 10 μM manganese chloride premixed solution

## References

[1] Sanders R., Huggett J.F., Bushell C.A., Cowen S., Scott D.J., Foy C.A. (2011). Evaluation of digital PCR for absolute DNA quantification. Anal. Chem..

[2] Pinheiro L.B., Coleman V.A., Hindson C., Herrmann J., Hindson B.J., Bhat S., Emslie K.R. (2012). Evaluation of a Droplet Digital Polymerase Chain Reaction Format for DNA Copy Number Quantification. Anal. Chem..

[3] Baker M. (2012). Digital PCR hits its stride. Nat. Method..

[4] Li J., Macdonald J., Von Stetten F. (2019). Review: a comprehensive summary of a decade development of the recombinase polymerase amplification. Analyst.

[5] Ma Y., Luo K., Chang W., Lee G. (2018). A microfluidic chip capable of generating and trapping emulsion droplets for digital loop-mediated isothermal amplification analysis. Lab chip.

[6] Wang R., Zhao R., Li Y., Kong W., Guo X., Yang Y. (2018). Rapid detection of multiple respiratory viruses based on microfluidic isothermal amplification and a real-time colorimetric method. Lab chip.

[7] Fu Y., Zhou H., Jia C., Jing F., Jin Q., Zhao J. (2017). A microfluidic chip based on surfactant-doped polydimethylsiloxane (PDMS) in a sandwich configuration for low-cost and robust digital PCR. Sensor. Actuator. B Chem..

[8] Kreutz J.E., Wang J., Sheen A.M., Thompson A.M., Staheli J.P., Chiu D.T. (2019). Self-Digitization Chip for Quantitative Detection of Human Papillomavirus Gene Using Digital LAMP. Lab chip.

[9] Liao P., Huang Y. (2017). Digital PCR: Endless Frontier of ‘Divide and Conquer’. Micromachines.

[10] Hindson B.J., Ness K., Masquelier D.A., Belgrader P., Heredia N.J., Colston B.W. (2011). High-Throughput Droplet Digital PCR System for Absolute Quantitation of DNA Copy Number. Anal. Chem..

[11] Feng Q., Gai F., Sang Y. (2018). A comparison of QuantStudio™ 3D Digital PCR and ARMS-PCR for measuring plasma EGFR T790M mutations of NSCLC patients. Cancer Manag. Res..

[12] Gou T., Hu J., Wu W., Mu Y. (2018). Smartphone-based mobile digital PCR device for DNA quantitative analysis with high accuracy. Biosens Bioelectron..

[13] Cao L., Cui X., Hu J., Li Z., Xu F. (2017). Advances in digital polymerase chain reaction (dPCR) and its emerging biomedical applications. Biosens. Bioelectron..

[14] Sreejith K.R., Ooi C.H., Jin J., Nguyen N. (2018). Digital polymerase chain reaction technology–recent advances and future perspectives. Lab chip.

[15] Yamaguchi S., Suzuki T., Inoue K., Azumi Y. (2015). DC-driven thermoelectric Peltier device for precise DNA amplification. Jpn. J. Appl. Phys..

[16] Liu W., Zhang M., Liu X., Ding X. (2017). A Point-of-Need infrared mediated PCR platform with compatible lateral flow strip for HPV detection. Biosens. Bioelectron..

[17] Li Y., Fu Y.Q., Brodie S.D., Walton A.J. (2012). Integrated microfluidics system using surface acoustic wave and electrowetting on dielectrics technology. Biomicrofluidics.

[18] Huang X., Elsayed M.A. (2010). Gold nanoparticles: Optical properties and implementations in cancer diagnosis and photothermal therapy. J. Adv. Res..

[19] Son J.H., Cho B., Hong S., Lee S.H., Hoxha O., Lee L.P. (2015). Ultrafast photonic PCR. Light. Sci. Appl..

[20] Son J.H., Hong S., Haack A.J., Gustafson L., Lee L.P. (2016). Rapid Optical Cavity PCR. Adv. Healthcare Mater..

[21] Roche P.J., Najih M., Lee S.S., Trifiro M. (2017). Real time plasmonic qPCR: how fast is ultra-fast? 30 cycles in 54 s. Analyst.

[22] Lee J., Cheglakov Z., Yi J., Cronin T.M., Weizmann Y. (2017). Plasmonic Photothermal Gold Bipyramid Nanoreactors for Ultrafast Real-Time Bioassays. J. Am. Chem. Soc..

[23] Vanzha E., Pylaev T., Khanadeev V. (2016). Gold nanoparticle-assisted polymerase chain reaction: effects of surface ligands, nanoparticle shape and material. RSC Adv..

[24] Yan L., Li J., Liu N., Hao X., Li D. (2017). Thermostable gold nanoparticle-doped silicone elastomer for optical materials. Colloids and Surfaces A: Physicochem. Eng. Asp..

[25] Wu W., Tracy J.B. (2015). Large-Scale Silica Overcoating of Gold Nanorods with Tunable Shell Thicknesses. Chem. Mater..

[26] Li H., Zhang H., Xu Y., Tureckova A. (2019). Versatile digital polymerase chain reaction chip design, fabrication, and image processing. Sensor. Actuator. B Chem..

[27] Pastorizasantos I., Kinnear C., Perezjuste J., Lizmarzan L.M. (2018). Plasmonic polymer nanocomposites. Nat. Rev. Mater..

[28] Notomi T., Okayama H., Masubuchi H.O., Yonekawa T., Hase T. (2000). Loop-mediated isothermal amplification of DNA. Nucleic Acid. Res..

[29] Gregorini M., Mikutis G., Grass R.N., Stark W.J. (2019). Small-Size Polymerase Chain Reaction Device with Improved Heat Transfer and Combined Feedforward/Feedback Control Strategy. Ind. Eng. Chem. Res..

[30] Joanicot M., Ajdari A. (2005). Droplet Control for Microfluidics. Science.

[31] Gansen A., Herrick A.M., Dimov I.K. (2012). Digital LAMP in a sample self-digitization (SD) chip. Lab chip.

